# Apparently X-linked Foveal Hypoplasia of Two Brothers: A Report of a Rare Case

**DOI:** 10.7759/cureus.53891

**Published:** 2024-02-09

**Authors:** Ghufran Alarfaj, Hassan Alhashim, Horia M Alotaibi, Mahdi Almubarak, Jinan Alhamad

**Affiliations:** 1 Ophthalmology, Dhahran Eye Specialist Hospital, Dhahran, SAU; 2 Ophthalmology, Imam Abdulrahman Bin Faisal University, Dammam, SAU; 3 Ophthalmology, Ministry of Health, Riyadh, SAU

**Keywords:** optical coherence tomography (oct), nystagmus, visual acuity, case report, foveal hypoplasia

## Abstract

Foveal hypoplasia is a retinal disorder characterized by the anatomic absence of the foveal pit. It might be isolated or associated with poor vision and several conditions such as albinism, aniridia, microphthalmos, congenital nystagmus, or other diseases. Genetic and non-genetic causes can play a role in foveal pit development. However, the exact mechanism that causes foveal pit absence has not been determined. This study reports a five-year-old boy who presented to the eye clinic with bilateral poor vision since birth. Optical coherence tomography (OCT) was performed and confirmed the absence of the foveal pit in both eyes. Diagnosis of foveal hypoplasia was made. The parents reported a positive family history of similar conditions, specifically, a paternal grandfather, a male paternal cousin, and a brother. To the best of our knowledge, this is the first reported case of foveal hypoplasia, with a positive family history in the male gender specifically. Thus, inheritance is presumed to be X-linked recessive. We acknowledge that further investigation by genetic testing would offer further insight into this case.

## Introduction

Normal development of the foveal layer in the retina starts at 25 weeks of gestational age and reaches maturity between 15 and 45 months after birth. The central pit is formed by the centrifugal movement of the inner retinal layers and the centripetal packing of cone photoreceptors. A disruption of any step of the foveal development process will lead to foveal hypoplasia. Foveal hypoplasia is a retinal pathology defined by the anatomic absence of the foveal pit. The foveal pit is a specialized structure developed from the central retina and referred to as the fovea centralis. Foveal hypoplasia might present alone or be associated with other congenital conditions [1].

Comparatively, fovea plana also identified by loss of foveal pit usually occurs in patients with good best-corrected visual acuity (BCVA) despite abnormal optical coherence tomography (OCT) findings. Approximately 3% of children with normal clinical examination of eyes had a structurally underdeveloped foveal pit on OCT bilaterally. Infrequent cases of unilateral fovea plana suggest that independent factors such as genetic mosaicism or local tissue environment might play a role in developing fovea plana [2,3].

We report a case of foveal hypoplasia in a five-year-old boy with a positive family history of similar conditions, specifically, a paternal grandfather, a male paternal cousin, and a brother, without clear associated conditions. It could be the first reported case of foveal hypoplasia with a positive family history in the male gender specifically, in which inheritance is presumed to be X-linked recessive. Another possibility is ocular albinism without usual hypopigmented iris and iris transillumination defects.

## Case presentation

Case 1

A five-year-old boy patient presented to the eye clinic at Dhahran Eye Specialist Hospital in 2019 complaining of bilateral decreased vision associated with abnormal eye movement since birth. There was no history of lighter color of skin during early childhood or irregular skin hypopigmentation. Medical and surgical ophthalmic history was unremarkable, except for glasses that he wears for fully accommodative esotropia. There was no history of trauma. He was medically free and not using any medications. His prenatal history was unremarkable, and he was born with full-term normal vaginal delivery. Furthermore, there was no neonatal intensive care unit (NICU) admission in the post-delivery period. Regarding family history, parents reported a positive family history of similar conditions, specifically, a paternal grandfather, a male paternal cousin, and a brother.

**Figure 1:**
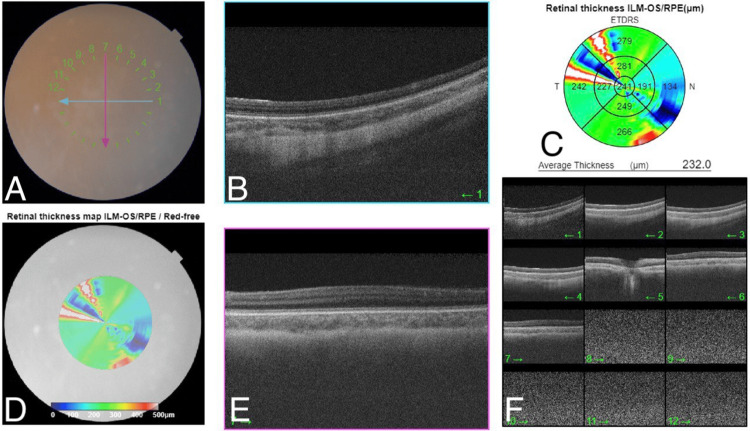
OCT of the right eye revealed the absence of the foveal pit with the persistence of inner retinal layers in the foveolar area. A: The color fundus photo shows a normal optic disc, normal vasculature, blunt fovea, and flat retina. B: The spectral domain macular OCT on 1 o'clock shows loss of foveal contour. C: The retinal thickness map shows normal central retinal thickness with some artifacts. D: The red free fundus photo shows a blunt fovea and flat retina. E: The spectral domain macular OCT on 7 o'clock shows loss of foveal contour. F: Multi-directions of OCT confirmed loss of foveal contour in all directions. OCT: optical coherence tomography

**Figure 2:**
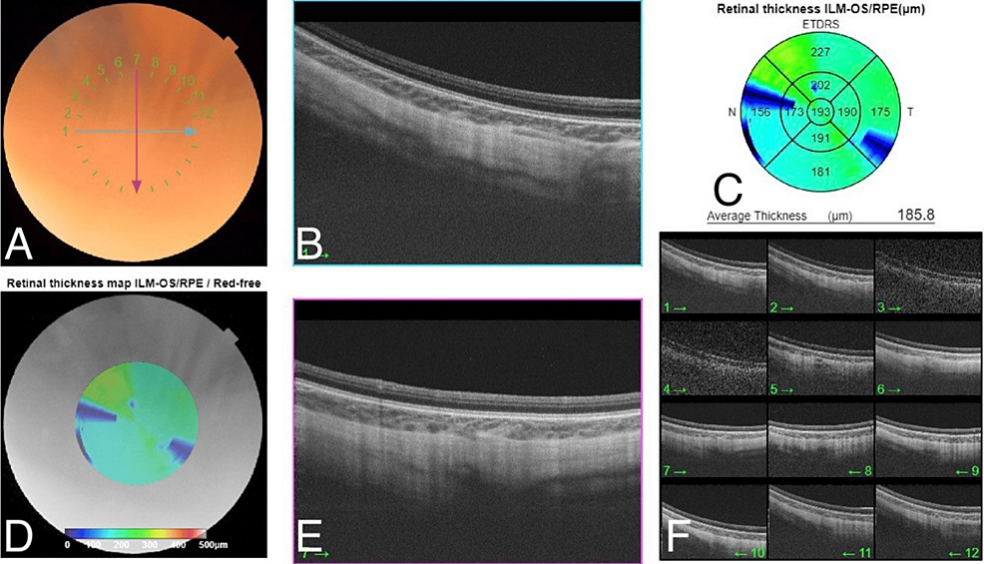
OCT of the left eye revealed the absence of the foveal pit with the persistence of inner retinal layers in the foveolar area. A: The color fundus photo shows a normal optic disc, normal vasculature, blunt fovea, and flat retina. B: The spectral domain macular OCT on 1 o'clock shows loss of foveal contour. C: The retinal thickness map shows normal central retinal thickness with some artifacts. D: The red free fundus photo shows a blunt fovea and flat retina. E: The spectral domain macular OCT on 7 o'clock shows loss of foveal contour. F: Multi-directions of OCT confirmed loss of foveal contour in all directions. OCT: optical coherence tomography

On examination, his BCVA was 20/200 in both eyes. Intraocular pressure was 15 mmHg in both eyes. Pupils were equal, round, regular, and reactive to light and accommodation. Slit-lamp examination revealed posterior embryotoxon without associated iris atrophy or corectopia. A dilated fundus examination showed pigmentary changes and blunt foveal reflex. Optic nerves and retinal periphery were normal, with no associated uveitis signs or degenerations. Cycloplegic refraction in the right eye was +6.50 -1.00 × 10° and in the left eye +6.50 -1.00 × 170°. OCT of both eyes revealed the absence of the foveal pit with the persistence of inner retinal layers in the foveolar area (Figure 1 and Figure 2).

**Figure 3:**
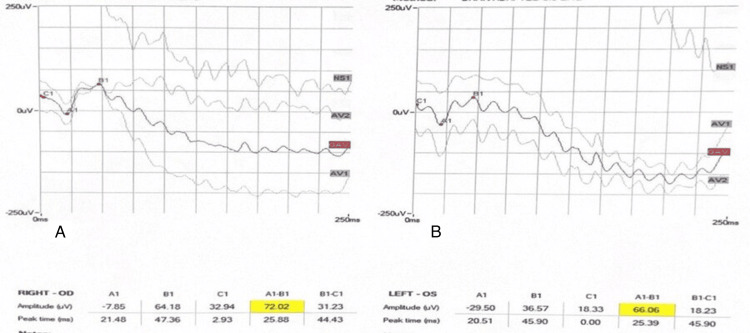
Dark-adapted 3.0 ERG of both eyes. A: OD right eye. B: OS left eye. ERG: electroretinogram

**Figure 4:**
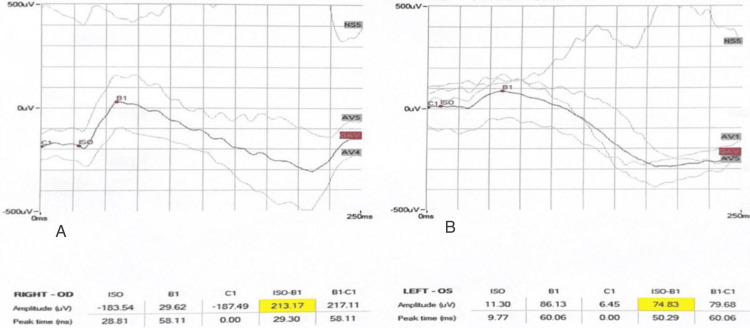
Dark-adapted 0.01 ERG of both eyes. A: OD right eye. B: OS left eye. ERG: electroretinogram

**Figure 5:**
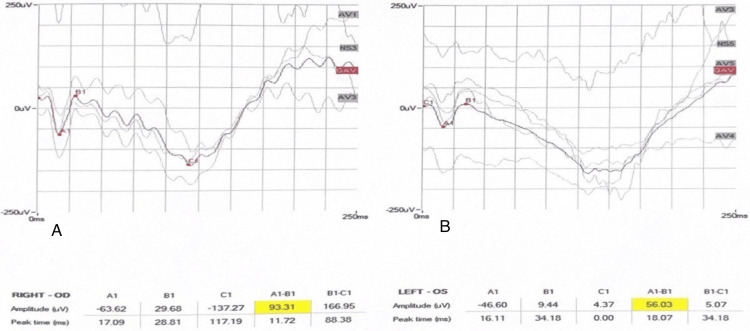
Dark-adapted 10 ERG of both eyes. A: OD right eye. B: OS left eye. ERG: electroretinogram

**Figure 6:**
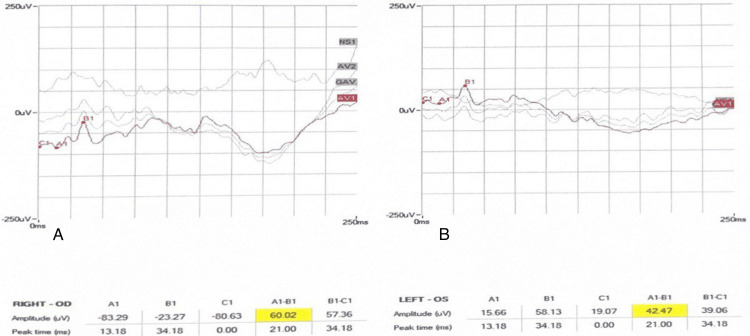
Light-adapted 3.0 ERG of both eyes A: OD right eye. B: OS left eye. ERG: electroretinogram

**Figure 7:**
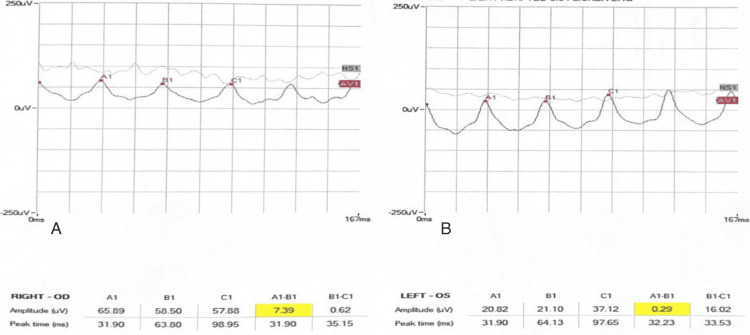
Light-adapted 3.0 FLICKER ERG of both eyes. A: OD right eye. B: OS left eye. ERG: electroretinogram

Electroretinogram (ERG) dark-adapted and light-adapted was performed, and the patient's findings were found to be normal (Figures 3-7).

Case 2

**Figure 8:**
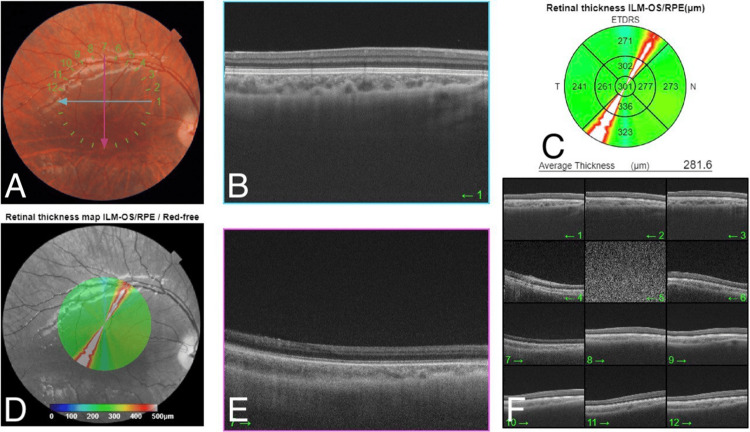
OCT of the right eye revealed the absence of the foveal pit. A: The color fundus photo shows a normal optic disc, normal vasculature, blunt fovea, and flat retina. B: The spectral domain macular OCT on 1 o'clock shows loss of foveal contour. C: The retinal thickness map shows normal central retinal thickness with some artifacts. D: The red free fundus photo shows a blunt fovea and flat retina. E: The spectral domain macular OCT on 7 o'clock shows loss of foveal contour. F: Multi-directions of OCT confirmed loss of foveal contour in all directions. OCT: optical coherence tomography

**Figure 9:**
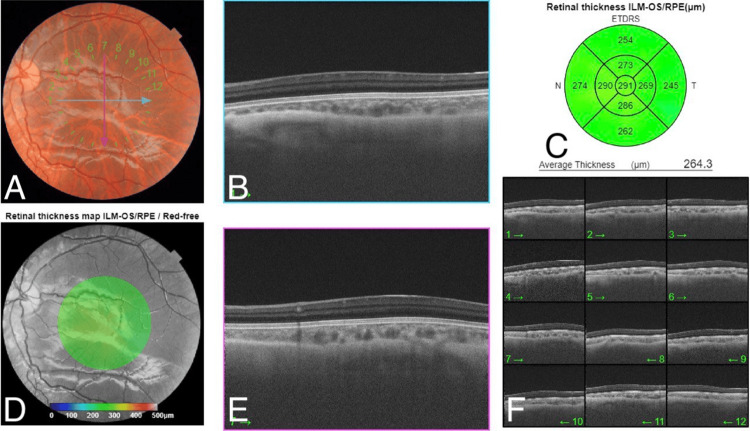
OCT of the left eye revealed the absence of the foveal pit. A: The color fundus photo shows a normal optic disc, normal vasculature, blunt fovea, and flat retina. B: The spectral domain macular OCT on 1 o'clock shows loss of foveal contour. C: The retinal thickness map shows normal central retinal thickness with some artifacts. D: The red free fundus photo shows a blunt fovea and flat retina. E: The spectral domain macular OCT on 7 o'clock shows loss of foveal contour. F: Multi-directions of OCT confirmed loss of foveal contour in all directions. OCT: optical coherence tomography

Moreover, the family brought his two-year-old brother who also complains of decreased vision since he was born. A review of his medical and surgical history revealed that he is medically and surgically free with no prenatal or postnatal complications or trauma. General observation shows the boy wears glasses There was no abnormal head position or any dysmorphic feature. Examination of vision was central, unsteady, and unmaintained in both eyes. Intraocular pressure was 15 mmHg in both eyes. Pupils were equal, round, regular, and reactive. Slit-lamp examination was within normal. Dilated fundus examinations of both eyes showed clear media and normal optic disc with a 0.3 cup-to-disc ratio; the macular area showed pigmentary changes and blunt foveal reflex. The retina looked flat with no breaks or detachment. Retinal vasculature was normal in size and configuration. There were no signs of any retinal degeneration or dystrophy. Cycloplegic refraction in the right eye was Plano -2.00 × 100° and in the left eye Plano -2.00 × 80°. ERG was done and was not suggestive of retinitis pigmentosa, which was similar to his brother's findings. OCT revealed the absence of the foveal pit (Figure 8 and Figure 9).

## Discussion

Foveal hypoplasia results from the disruption of any step of the fovea retinal development processes [1]. It is known that genetic and non-genetic causes can play a role in foveal pit development. However, the exact mechanism that causes foveal pit absence has not been determined. Before the advent of OCT, foveal hypoplasia has been detected by ophthalmoscopy, fluorescein angiography, and histology [1]. Currently, OCT remains the best non-invasive and quick method to investigate foveal hypoplasia, especially in those patients with unexplained impaired vision [2]. Decreased vision varies among patients who lack a foveal pit. It is suggested by Marmor et al. [4] that the absence of the foveal pit doesn't necessarily lead to decreased vision as the cone function can be preserved despite the pit. Thomas et al. [5] were able to establish a grading system of foveal hypoplasia based on OCT findings that could be correlated with the decrease in visual acuity. Grading was based on the presence or absence of four structural features: (a) extrusion of plexiform layers, (b) foveal pit, (c) outer segment (OS) lengthening, and (d) outer nuclear layer (ONL) widening. Also, they found a strong association between visual acuity and each grade as well. Our patients lacked a foveal pit, with OS lengthening and ONL widening. In addition, there was perseverance of all the inner retinal layers and outer plexiform layer which makes it a grade 2 according to Thomas et al.'s classification with a visual acuity of 20/200. Foveal hypoplasia can present with or without identifiable gene mutation [6]. Additionally, foveal hypoplasia might be isolated or associated with several disorders such as albinism, aniridia, microphthalmos, congenital nystagmus, or other conditions. Foveal hypoplasia is labeled typical when associated with PAX6 mutation, albinism, or retinopathy of prematurity and atypical when associated with achromatopsia [7-9]. When PAX6 mutation is associated with foveal hypoplasia and normal irides, it is usually autosomal dominant unlike the inheritance in our case which is assumed to be X-linked recessive [7].

Diversely, X-linked foveal hypoplasia is more linked to ocular albinism specifically GPR143 (MIM 300808) mutations and Aland Island eye disease which is caused by CACNA1F (MIM 300110) mutations [6]. Aland Island eye disease (known as ocular albinism 2) is associated with color vision defects. However, color vision testing was not performed considering the young age of the patients. Alternatively, ERG was conducted, and it tends to have a negative b-wave in scotopic ERG which is not consistent with the findings encountered in our patient's ERG [10-12]. On the other hand, the GPR143 mutations that are believed to be causing the Nettleship-Falls ocular albinism (known as ocular albinism 1) phenotypically can present with normal-colored iris as reported by Charles et al. [13,14]. In ERG exam, those patients tend to have normal or supranormal ERG as in our case [15]. Other associations in the literature found in our patients were hyperopic refractive error, posterior embryotoxon, and strabismus [16]. Interestingly, ERG testing conducted by Mao et al. [17] in one Chinese family that had the GPR143 mutations was found to give attenuated b-wave in full-field ERG unlike our case and previous literature. Another differential diagnosis is mentioned by Takkar et al. [18] who reported X-linked foveal hypoplasia in a patient with dyschromatosis universalis hereditaria. However, it is unlikely in our patients because they lack dermal pigmentary anomalies.

## Conclusions

We present a rare case of foveal hypoplasia with a positive family history among males specifically indicating an X-linked recessive inheritance. The cases could be isolated X-linked foveal hypoplasia or more likely a case of ocular albinism type 1 with dark-colored hair and irides which has not been reported before in the Kingdom of Saudi Arabia. However, there is still the need to do genetic testing.
